# Investigation of antioxidant, antimicrobial and toxicity activities of lichens from high altitude regions of Nepal

**DOI:** 10.1186/s12906-017-1797-x

**Published:** 2017-05-25

**Authors:** Baidya Nath Jha, Mitesh Shrestha, Durga Prasad Pandey, Tribikram Bhattarai, Hari Datta Bhattarai, Babita Paudel

**Affiliations:** 1 Research Institute for Bioscience and Biotechnoogy, RIBB, Sinamangal, Kathmandu, Nepal; 2 Central Department of Biotechnology, Tribhuvan University, Kirtipur, Kathamndu Nepal; 3 Research Center for Applied Science and Technology, Tribhuvan University, Kirtipur, Kathmandu Nepal

**Keywords:** Antimicrobial, Antioxidant, DPPH, Lichen, Thin layer chromatography

## Abstract

**Background:**

Several lichen species are reported to be used tradiationally in many theraupatic practices. Many lichen species are reported as sources of several bioactive natural compounds. Several lichen species of Nepal are so far chemically unexplored.

**Methods:**

The morphological, anatomical and phytochemical characteristics of lichens were compared for the taxonomic identification of the species. Methanol- water extract of lichens were sub fractionated into hexane, dichloromethane and methanol fractions for bioactivity assays. Antimicrobial activities of extracts were evaluated agaisnt pathogenic bacteria and fungal species. DPPH test was used for antioxidant potential evaluation. Brineshrimp test was perfermed to evaluate toxicity of the extracts.

**Results:**

A total of 84 lichen specimens were collected and identified from Annapurna Conservation Area (ACA) Nepal. The specimens were identified as belonging to 19 genera and 47 species. Methanol fractions of 16 specimens and dichloromethane (DCM) fractions of 21 lichens specimens showed antioxidant activities comparable with commercial standards (BHA, Butylated hydroxyanisole, IC50=4.9±0.9 μg/mL) even at crude extract level. Similarly, the DCM fraction of 17 lichens showed potential antimicrobial activity against a Gram-positive bacterium (Staphylococcus aureus KCTC3881) and DCM fractions of 45 lichens showed antimicrobial activity against a Gram-negative bacterium (Klebsiella pneumoniae KCTC2242). DCM fractions of three lichens showed antifungal activity against the yeast, Candida albicans KCTC 7965. Likewise, methanol fractions of 39 lichens and DCM fractions of 74 lichens showed strong toxicity against brine shrimp nauplii with more than 80% mortality.

**Conclusion:**

Such biological activity-rich lichen specimens warrant further research on exploration of natural products with antioxidant, antimicrobial and anti cancer (toxic) potential.

## Background

Lichens are cosmopolitan in distribution from arctic to tropical regions and from the plains to the highest mountains, and some survive in the extreme environment of deserts. Lichen metabolites of several chemical classes such as - aliphatic acids, pulvinic acid derivatives, depsides and depsidones, dibenzofurans, diterpenes, anthraquinones, naphthoquinones, xanthones, and epidithiopiperazinediones have been described. These compounds have shown a wide range of biological activities such as antibiotic, antimycobacterial, antiviral, anti-inflammatory, analgesic, antipyretic, antiproliferative, cytotoxic effects and antioxidant properties [1, 2].

Several pathogenic bacteria have developed into multiple drug resistant strains. To overcome such phenomenon, several novel antibacterial compounds need to be developed. A number of lichens have been screened for antibacterial activity [3]. Several lichen compounds were found active against Gram-positive bacteria and mycobacteria [4].

Reactive oxygen species (ROS) are harmful for living organisms [5] causing chronic inflammation, which in turn can lead to several diseases including cardio-vascular diseases, cancer, age-related disorders, metabolic disorders, and atherosclerosis [6]. ROS react with various cellular components including DNA and ultimately leading to cell inactivation or death [7]. Living organisms accumulate various ROS through both normal metabolic processes and exogenous sources like environmental stresses. Various types of ROS, e.g. hydrogen peroxide (H_2_O_2_) are produced inside the living organisms [8]. These ROS are highly reactive and unstable due to the presence of an unpaired electron in their outer shell. The administration of antioxidants can remove or reduce the content of ROS in human body and thereby reduce the negative impacts of ROS in human health. Most of the knowledge on antioxidant activities of lichens and their chemical investigations are derived from the species of tropical and sub-tropical origin. Fewer studies have been conducted on the antioxidant activities of lichens from extreme environments such as the Antarctic regions [9, 10] and high altitude region of Nepal [11].

Nepal is a mountainous country which contains diverse geographic locations ranging from 60 m to 8848 m above sea level (asl). In Nepal, 465 species of lichens from 79 genera have been recorded, of which 48 species are described as endemic [12]. Because of its unique bio-geographic location, altitudinal variation and diverse climatic and topographic conditions, several lichen species can be obtained with in a small geographical area. Traditionally, a variety of lichen species are used to prepare medicines for several diseases [13]. There are very few reports of chemical investigations of lichens species from Nepal [2]. In this report we investigated antioxidant, antibacterial, antifungal and toxic activities of lichens from the Annapurna Conservation Area (ACA) region. The phytochemistry of those specimens is yet to be explored.

## Methods

### Collection and identification of lichen species

A total of eighty four lichen specimens (Table 1) were collected from five different geographical locations of ACA of western Nepal in the time period of Janauary, 2014 to April, 2014. The number of specimens collection per collection site was as follows: Chame (2713 m asl), Danaque (2230 m asl), Jomsom (2760 m asl), Ghandruk (2012 m asl) and Sarangkot (1750 m asl). All the lichen specimens were identified by analyzing morphological, anatomical and phytochemical characteristics using a lichen identification check list [14]. Among others, twenty two specimens were identified up to genus level only. Voucher specimens of the lichen are deposited in the lichen herbarium of the Research Institute of Bioscience and Biotechnology, Kathmandu, Nepal.

**Table 1:** Lichens collected from Annapurna conservation area and their biological activities

S.N	Symbol	Name	Dry wt.	Activity DPPH^a^		Anti microbial activity	Brine shrimp		*Candida albicans*(mm)
				Methanol	DCM	DCM	MeOH	DCM	DCM fraction
1	DAN-1	*Parmotrema cetratum*	10	44.4 ± 0.5	11.4 ± 0.1	Α (7) B (7)	S	S	-
2	DAN-2	*Usnea pectinata*	10	-	-	-	S	S	-
3	DAN-3	*Ramalina conduplicans*	10	-	22.9 ± 0.7	Β(12)	S	S	-
4	DAN-4	*Everniastrum cirrhatum*	2	-	-	Β(11)	I	S	-
5	DAN-5	*Parmotrema sanoti-angelii*	10	-	-	A(10) B(10)	S	S	-
6	DAN-6	*Parmotrema reticulatum*	10	23.3 ± 0.3	22.1 ± 0.1	A(10) B(9)	S	S	-
7	DAN-7	*Heterodermia diademata*	10	-	-	Β (7)	I	S	-
8	DAN-8	*Cladonia verticillata*	9.1	-	44.1 ± 0.3	Β (8)	W	S	-
9	DAN-9	*Parmotrema* sp.	10	-	-	Β (7)	I	S	-
10	DAN-10	*Parmotrema tinctorum*	10	-	-	Β (7)	I	S	-
11	DAN-11	*Peltigera polydactyla*	3.6	11.3 ± 0.2	19.6 ± 0.1	-	I	S	--
12	JOM-1	*Lobaria retigera*	10	-	-	-	S	S	-
13	JOM -2	*Parmotrema thomsonii*	10	-	-	Α(8) B (11)	S	S	-
14	JOM -3	*Ramalina* sp.	10	-	-	-	S	S	-
15	JOM -4	*Everniastrum cirrhatum*	10	-	-	Β(14)	S	S	-
16	JOM -5	*Usnea* sp.	10	-	-	Β(10)	S	S	-
17	JOM -6	*Peltigera polydactyla*	9.2	11.2 ± 0.1	-	-	S	S	-
18	JOM -7	*Heterodermia diademata*	10	-	-	-	I	S	12
19	JOM -8	*Parmotrema tinctorum*	10	-	-	Α(11) B(7)	S	S	-
20	JOM -9	*Leptogium delavayi*	10	-	-	-	W	I	-
21	JOM -10	*Parmotrema reticulatum*	5.9	-	-	-	S	I	-
22	JOM -11	*Parmelina quercina*	5.6	-	-	Β(10)	I	S	-
23	JOM -12	*Cladonia squamosa*	10	-	-	Α(12)B(13)	I	S	-
24	JOM -13	*Parmotrema* sp.	10	-	-	Α(11)B(7)	S	S	14
25	JOM -14	*Heterodermia indica*	2.8	-	49.9 ± 2.4	Α(9)B(8)	I	S	11
26	JOM -15	*Heterodermia leucomela*	3.8	-	47.7 ± 0.5	Α(8)B(9)	I	S	-
27	JOM -16	*Leptogium delavayi*	0.9	-	47.1 ± 1.8	-	I	W	-
28	CHA-1	*Ramalina roesleri*	10	-	20.2 ± 0.2	-	I	S	-
29	CHA-2	*Parmotrema* sp.	10	-	-	-	S	I	-
30	CHA-3	*Parmotrema* sp.	10	-	-	-	S	S	-
31	CHA-4	*Leptogium* sp.	0.8	-	-	-	I	S	-
32	CHA-5	*Cladonia verticillata*	10	-	-	-	I	S	-
33	CHA-6	*Usnea* sp.	9	-	-	-	S	S	-
34	CHA-7	*Lepraria* sp.	6.8	-	-	-	S	I	-
35	CHA-8	*Peltigera polydactyla*	10	5.7 ± 0.02	5.56 ± 0.2	-	I	S	-
36	CHA-9	*Lepraria* sp.	10	-	-	-	I	W	-
37	GHAN-1	*Coccocarpia erythroxyli*	3.4	-	-	-	I	I	-
38	GHAN-2	*Parmotrema melanothrix*	10	-	-	Β(7)	S	S	-
39	GHAN-3	*Heterodermia diademata*	10	-	-	Β(7)	W	S	-
40	GHAN-4	*Parmotrema praesorediosum*	10	-	-	Β(9)	I	S	-
41	GHAN-5	*Pertusaria leucosora*	10	22.4 ± 0.9	44.6 ± 1.0	Β(13)	S	S	-
42	GHAN-6	*Cladonia* sp.	10	-	-	Β(9)	I	S	-
43	GHAN-7	*Heterodermia diademata*	10	-	-	-	S	S	-
44	GHAN-8	*Heterodermia punctifera*	10	43.9 ± 2.1	47.3 ± 0.6	-	S	S	-
45	GHAN-9	*Parmotrema* sp.	3	22.8 ± 0.9	-	Β(12)	W	S	-
46	GHAN-10	*Leptogium delavayi*	8.5	-	-	-	W	I	-
47	GHAN-11	*Lecidea* sp.	10	-	-	Β(12)	S	S	-
48	GHAN-12	*Parmelia* sp.	10	-	-	Β(9)	S	S	-
49	GHAN-13	*Parmotrema* sp.	10	19.9 ± 3.0	-	Β(11)	I	S	-
50	GHAN-14	*Collema* sp.	1.7	-	-	-	I	I	-
51	GHAN-15	*Heterodermia microphylla*	10	-	23.7 ± 0.1	-	I	S	--
52	GHAN-16	*Parmelia meiophora*	6.9	-	-	Β(15)	S	S	-
53	GHAN-17	*Usnea baileyi*	1.8	-	-	-	S	S	-
54	GHAN-18	*Heterodermia leucomelos*	10	-	-	Α(7)B(7)	I	S	-
55	GHAN-19	*Parmotrema* sp.	10	-	-	Β(11)	S	S	-
56	GHAN-20	*Pannaria complanata*	10	-	-	Α(11)B(10)	S	S	-
57	GHAN-21	*Hypotrachyna flexilis*	10	-	-	Β(9)	I	S	-
58	SAR-1	*Heterodermia indica*	7.8	21.2 ± 0.3	24.0 ± 0.1	Β(8)	S	S	-
59	SAR-2	*Parmotrema reticulatum*	10	-	-	Β(9)	S	S	-
60	SAR-3	*Heterodermia punctifera*	7.83	-	-	-	I	S	-
61	SAR-4	*Usnea coralline*	9.6	-	-	-	S	S	-
62	SAR-5	*Ramalina conduplicate*	5.3	-	49.0 ± 2.3	Β(12)	S	S	-
63	SAR-6	*Lobaria disecta*	10	-	50.7 ± 2.2	Β(7)	W	S	-
64	SAR-7	*Parmotrema reticulatum*	10	-	-	Β(7)	W	S	-
65	SAR-8	*Heterodermia podocarpa*	10	-	-	Β(12)	W	S	-
66	SAR-9	*Parmelaria thomsonii*	10	87.3 ± 7.1	-	Β(10)	W	S	-
67	SAR-10	*Everniastrum nepalenses*	10	-	-	Β(8)	S	S	-
68	SAR-11	*Cladonia coccifera*	10	23.5 ± 0.8	-	-	I	S	-
69	SAR-12	*Parmotrema reticulatum*	5.7	-	53.1 ± 6.9	-	S	S	-
70	SAR-13	*Parmotrema* sp.	10	47.8 ± 1.3	-	Α(9)	S	S	-
71	SAR-14	*Parmelia omphalodes*	10	-	-	Α(10)B(11)	I	S	-
72	SAR-15	*Parmotrema reticulatum*	3.5	49.8 ± 0.1	-	Β(9)	M	S	-
73	SAR-16	*Parmotrema* sp.	1.5	-	-	Β(8)	S	S	-
74	SAR-17	*Heterodermia speciosa*	6	-	-	Α(7)	M	S	-
75	SAR-18	*Parmotrema* sp.	8.6	49.9 ± 0.3	-	-	S	S	-
76	SAR-19	*Parmotrema* sp.	8.4	-	-	Α(9)	S	S	-
77	SAR-20	*Heterodermia microphylla*	10	-	-	Α(8)	W	S	-
78	SAR-21	*Heterodermia microphylla*	7.3	-	-	-	I	I	-
79	SAR-22	*Parmelia omphalodes*	10	-	-	-	S	S	-
80	SAR-23	*Heterodermia microphylla*	6.4	-	24.1 ± 0.4	-	W	S	-
81	SAR-24	*Heterodermia microphylla*	1.2	-	45.8 ± 1.1	Α(9)	I	S	-
82	SAR-25	*Cladonia coccifera*	0.6	-	47.9 ± 0.2	-	I	S	-
83	SAR-26	*Heterodermia speciosa*	1.4	-	46.7 ± 1.3	Β(13)	I	S	-
84	SAR-27	*Pertusaria* sp.	0.4	49.3 ± 2.6	-	Β(12)	I	S	-
		BHA		5.0 ± 0.4					
		Ampicillin(10 μg)				A(20),B(19)			
		Amphotericin B(10 μg)							20
		Berberine chloride (8 μg)					W		
		Berberine chloride (12 μg)					M		
		Berberine chloride (16 μg)					S		

^a^IC_50_ (50% inhibition in DPPH color) data, A-antibacterial active against *Staphylococcus aureus* (inhibition zone in mm), B-antibacterial activity against *Klebsiella pneumoniae* (inhibition zone in mm), S-strong activity (more than 80% death of brine shrimp), M-Moderate(50%–80% of death of brine shrimp), W-Weak (less than 50% of death of brine shrimp), I-inactive (no death at all)

### Extraction

Completely freeze-dried and ground lichen samples, 1–10 g (Table 1) were extracted in a mixture of methanol and water (90:10 *v*/v). Then, the extracts were filtered and solvent was evaporated at 38 °C under vacuum. This extraction procedure was repeated three times to ensure the complete extraction of extractable compounds. The total extract of a specimen was dissolved in distilled water (100 mL) and washed with hexane (300 mL) three times to remove pigments and fats. The water phase of extract was further washed with dichloromethane (300 mL) (DCM) thrice to remove medium polar compounds. The remaining water phase was freeze-dried, and residue dissolved in methanol. Insoluble parts of sugar and polysaccharides were removed. The DCM and methanol fractions of the extracts were then lyophilized and stored at −20 °C until further use.

### Chemical reagents

2, 2-Diphenyl-1-picrylhydrazyl (DPPH), Butylated hydroxyanisole (BHA), Ampicillin, Amphotericin B and Berberine chloride were purchased from Sigma-Aldrich, USA. All chemicals and reagents were of analytical grade. The solvents used during extraction and chromatography were of normal grade quality and they were distilled before use. The bacterial culture media, nutrient agar (NA) and Nutrient Broth (NB) and the fungal growth medium, Potato dextrose medium (PDA) were purchased from Difco, USA.

### Antimicrobial assay

#### Target microorganisms and culture condition

Three clinical microorganisms, including one Gram-positive *Staphylococcus aureus* KCTC3881) and one Gram-negative (*Klebsiella pneumoniae* KCTC2242) bacteria and a fungus, *Candida albicans* KCTC 7965, were purchased from Korean Collection of Type Culture (KCTC). Bacterial strains were grown on NA at 37 °C and *C. albicans* was grown on PDA at 25 °C.

#### Disk diffusion assay

Antimicrobial testing was carried out by using a previously described paper disk assay [11]. Sterile paper disks (Adventic, Japan) of 6 mm size were loaded with lichen extract at a concentration of 2 mg/disk in triplicate and allowed to dry at room temperature under sterile conditions. The disks were kept on the surface of NA (Nutrient agar) or PDA (Potato dextrose agar) plates, which had been freshly swabbed with the overnight grown broth culture of the target microbial strains. Then, the plates were incubated at optimum growth temperature of each strain for 24–48 h. The zones of inhibition around the lichen extract loaded paper disks were reflective of the antimicrobial effectiveness of the extract. Paper disks loaded with methanol, the solvent used to dissolve crude extract, were used as negative controls and the paper disks loaded with Ampicillin and Amphotericin B were used as positive controls for bacteria and yeast respectively.

#### Antioxidant assay

##### DPPH free radical scavenging assay

Free radical scavenging activity for the lichen extracts was estimated by using a previously described method [15]. One mL of DPPH solution (0.1 mM of DPPH in methanol) was mixed with 3 mL of various concentrations of the test extract. The mixture was incubated at room temperature (RT) for 30 min and the absorbance was measured at 517 nm in a UV-Visible spectrophotometer (SCINCO). Reaction mixtures without the test extract and with (Butylated hydroxyanisole) BHA were used as negative and positive controls, respectively. The experiment was conducted in triplicate.

##### Brine shrimp lethality test

Brine shrimp lethality test (BST) was used to evaluate the toxicity of various lichen’s crude extracts [16] with slight modification. The eggs of *Artemia salina* were hatched in aerated seawater in light at 25 °C. The hatched active nauplii were attracted towards the direction of light. The active nauplii (about 100) were selected and treated with 1 mg/mL of the test samples in 24 well plates. The effects of the test samples were monitored after 24 h of treatment by observing the live nauplii. The mortality rate of the nauplii indicated the toxicity of the test samples. The activity was categorized into four groups-strong (80% to 100% death of nauplii), moderate (50% to 80% death of nauplii), weak (less than 50% death of nauplii) and inactive (no death at all) (Table 1). Berberine chloride was used as positive control and sea water was used as negative control.

##### TLC based antioxidant activity screening of lichen extracts

Thin layer chromatography analysis of all lichen extracts was performed by using analytical silica gel TLC plates (Merck). An aliquot of 200 microgram lichen extract was loaded in the bottom of TLC plates (size. 10 cm X 20 cm) and run on the mobile phase of 10% methanol in DCM. The plates were observed under UVs (254 nm and 365 nm) to observe the bands of separated compounds. The plates were sprayed with DPPH solution (4 mM) in methanol. The yellow color developed against purple surface background of DPPH indicated antioxidant active compounds in the extracts.

##### Data analysis

All data were expressed as mean ± SD from a minimum of three replicates. 50% Inhibition concentration (IC_50_) was calculated by using Microsoft Excel 2007.

## Results and discussion

### Collection and identification of lichens

A total of 84 lichen specimens comprising 19 genera and 47 species were collected from five different locations of ACA starting from the altitude of 1750 m asl to 2760 m asl.

### Antioxidant activity

In this experiment, in-vitro antioxidant assays based on electron transfer (ET) or hydrogen atom transfer (HAT) system- DPPH free radical was used to investigate the antioxidant activity of lichen extracts. ET-based assays measured the capacity of an antioxidant which reduces an oxidant by changing the color [15]. Thus, the degree of color change was correlated with antioxidant potential.

DPPH is commercially available stable free radical in aqueous or methanol solution and becomes a stable molecule by accepting an electron or hydrogen radical from antioxidant compounds [15]. All the tested lichen extracts and the commercial standard (BHA) exhibited DPPH free radical scavenging capacity in the concentration dependent manner. BHA is a strong commercial antioxidant agent and the IC_50_ of this compound was determined as 4.98 ± 0.4 μg/mL.

In the present experiment, the test lichens extract showed the varying strengths to scavenge DPPH free radical. Overall, the IC_50_ of DPPH free radical scavenging capacity of active lichens extract was found between 5.6 ± 0.2 to 87.3 ± 7.1 μg/mL (Table 1). The Table 1 shows that only 30 specimens’ extract possessed antioxidant activities. Among them, the DCM fractions of *Parmoterma centratum*, *Peltigera polydactyla* and *Ramalina roesleri* and methanol fractions of *Peltigera polydactyla* and *Parmoterma* sp. showed comparatively strong DPPH reducing activity. The methanol extract of *Parmoterma* sp. from Nepal origin showed stronger antioxidant activity (IC_50_, 11.4 ± 0.1 μg/ml) than the same species collected from Malaysia (IC_50_, >500 μg/ml) [17]. *Ramalina bourgeana* Mont. ex Nyl. (Ramalinaceae) is consumed as a folk medicine for diuretic and stone–dissolving properties [18]. In the present experiment the data showed that 16 specimens’ methanol fraction, 21 specimens’ DCM fraction, 7 specimens’ both methanol and DCM fractions, 9 specimens’ only methanol fractions and 14 specimens’ only DCM fractions were antioxidant active. The results indicated the variability of antioxidant compounds in the lichen extracts.

### Antimicrobial activities

Only DCM fraction of the lichen extracts were active against *S. aureus* and *K. pneumoniae*. The sizes of zone of inhibition of active fractions are given in Table 1. Seventeen extracts showed activities against *S. aureus* and 45 extracts showed activities against *K. pneumoniae*. Twelve extracts showed antibacterial activities against both *S. aureus* and *K. pneumoniae.* Thus, the test lichens were found to be antimicrobial active against both Gram-positive and Gram-negative bacterial strains. Only three specimens’ extract were active against *C. albicans*. The comparative study of obtained results (Table. 1) showed that the antimicrobial constituents in the lichens extracts were different.

### Toxicity against brine shrimp (*Artemia salina*)

Hundred percent of *Artemia* nauplii were alive after 24 h of experiment in the negative control. LC_50_ of positive control sample was obtained as 8.4 μg/mL. Interestingly, majority of the lichens extracts showed toxicity against Brine shrimp nauplii (Table 1). Four categories of activities have been observed: (S) strong-100-80% death of nauplii, (M) moderate-80-50% death of nauplii, (W) weak-less than 50% death and (I) inactive-no death of brine shrimp nauplii after 24 h of incubation. DCM fractions were found to be more toxic than methanol fractions. Strong activities were shown by 74 DCM fractions and 39 methanol fractions while 8 DCM fractions and 32 methanol fractions did not show any activity against *A. salina* nauplii. The lichen extracts of ACA region showed stronger toxicity against *A. salina* than the species of other region [11].

### TLC based antioxidant activity screening of lichen extracts

**Fig. 1:**
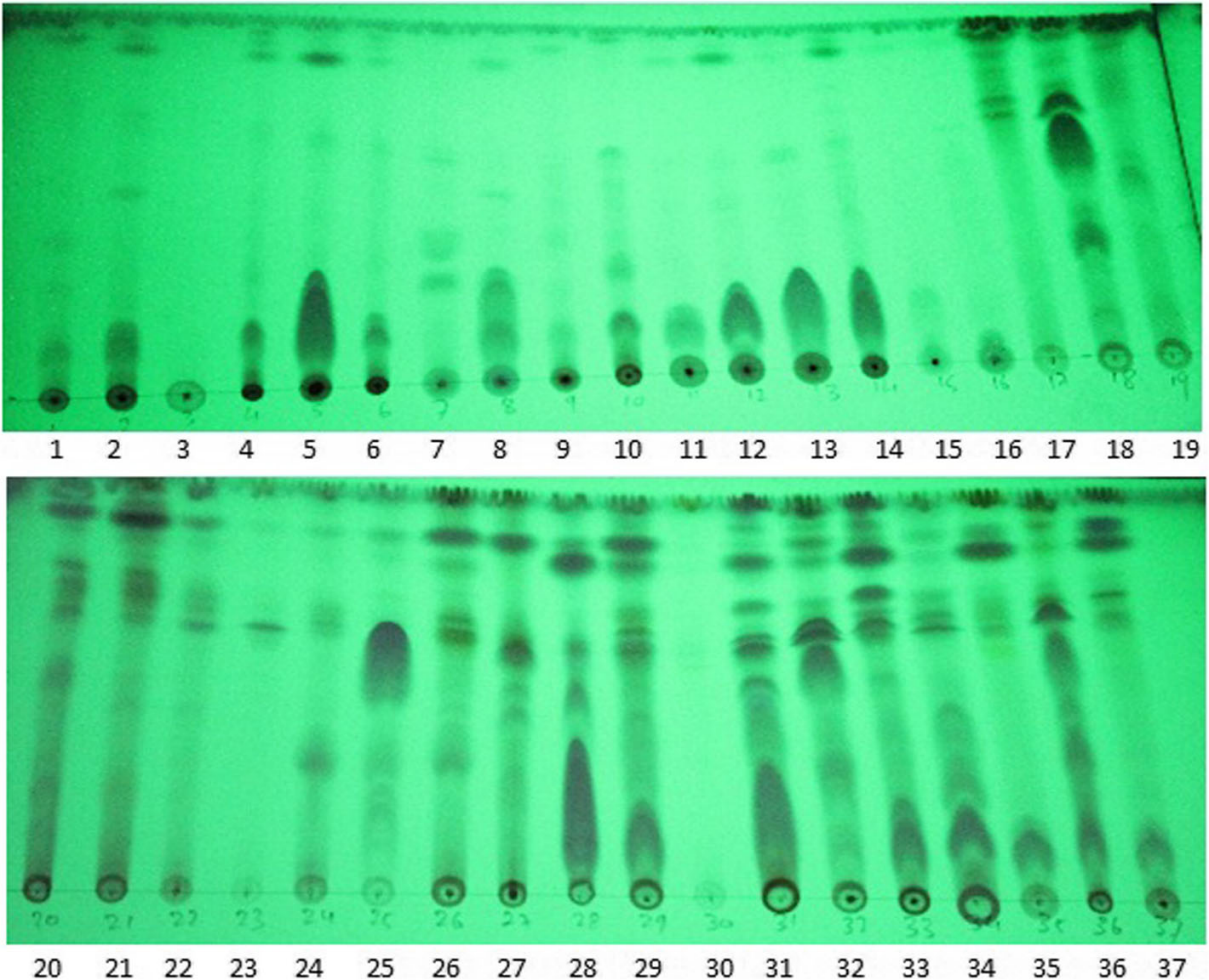
TLC based chemical screening of lichen extracts. Plate viewed under UV (254 nm). Mobile phase for TLC development was 10% methanol in DCM. W-methanol soluble water fraction, D-DCM fractions. The sample in the TLC plate is as follows: 1, SAR1W; 2, SAR9W; 3, SAR11W; 4, SAR13W; 5, SAR15W; 6, SAR18W; 7, SAR27W; 8, GHAN5W; 9, GHAN8W; 10, GHAN9W; 11, GHAN13W; 12, JOM6W; 13, DAN1W; 14, DAN6W; 15, DAN11W; 16, CHA8W; 17, SAR1D; 18, SAR5D; 19, SAR6D; 20, SAR12D; 21, SAR23D; 22, SAR24D; 23, SAR25D; 24, SAR26D; 25, GHAN5D; 26, GHAN8D; 27, GHAN15D; 28, JOM14D; 29, JOM15D; 23, JOM16D; 31, DAN1D; 32, DAN3D; 33, DAN6D; 34, DAN8D; 35, DAN11D; 36, CHA1D; 37 CHA8D

TLC based chemical screening of the bioactive fractions of the lichens showed a number of bioactive compounds contained in the extracts. As expected, compounds from methanol fractions had a lower Rf value than those from the DCM fraction in this TLC system, which indicates that they were more polar (Fig. 1). TLC based phytochemical screening was performed only for the extracts which showed antioxidant and antimicrobial activities.

**Fig. 2:**
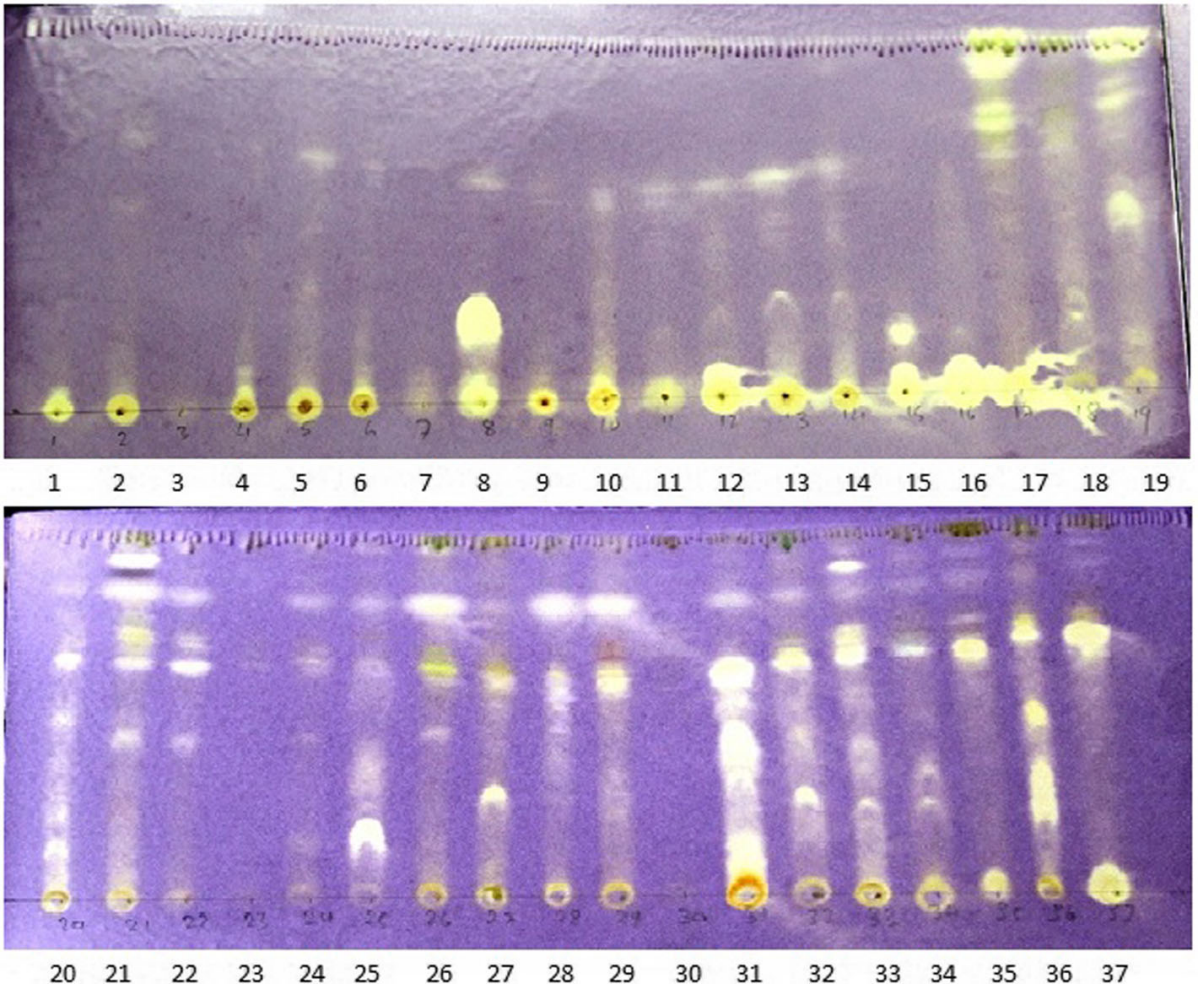
TLC based antioxidant screening of the active extracts. Mobile phase for TLC development was 10% methanol in DCM. The purple color was from DPPH and antioxidant active fractions showed reduced in purple color to yellow. The darkness of color spots indicated the potential of antioxidant activity, the size of color spot indicated the content of antioxidant active compounds in the extracts. W- methanol soluble water fraction, D- DCM fractions of lichen extracts. The sample in the TLC plate is as follows:1, SAR1W; 2, SAR9W; 3, SAR11W; 4, SAR13W; 5, SAR15W; 6, SAR18W; 7, SAR27W; 8, GHAN5W; 9, GHAN8W; 10, GHAN9W; 11, GHAN13W; 12, JOM6W; 13, DAN1W; 14, DAN6W; 15, DAN11W; 16, CHA8W; 17, SAR1D; 18, SAR5D; 19, SAR6D; 20, SAR12D; 21, SAR23D; 22, SAR24D; 23, SAR25D; 24, SAR26D; 25, GHAN5D; 26, GHAN8D; 27, GHAN15D; 28, JOM14D; 29, JOM15D; 23, JOM16D; 31, DAN1D; 32, DAN3D; 33, DAN6D; 34, DAN8D; 35, DAN11D; 36, CHA1D; 37, CHA8D

The number of antioxidant active bands in methanol fractions is comparatively small than in DCM fractions (Fig. 2). But, the intensity of color change of sprayed DPPH was much higher in the bands obtained from the methanol fractions than from the DCM fractions. The results indicated that water fractions contained stronger antioxidant compounds than the DCM fractions. The results also indicated that the DCM fractions contained higher number of moderately active antioxidants than the methanol fractions.

## Conclusion

A total of 84 lichen specimens including 19 genera and 47 species were collected and tested for antioxidant, antibacterial, antifungal and toxic properties against Brine shrimp nauplii. The test extract showed potent antioxidant activities. Methanol fractions were found to contain strong antioxidant compounds than DCM fractions as indicated in strong spots in TLC analysis plate after DPPH spray. The TLC analysis also indicated that DCM fractions contained the bigger number of antioxidant compounds. Similarly, several lichen specimens were found antibacterial active against the Gram-negative test strain *K. pneumoniae*. Most of the lichen extracts were found toxic against Brine shrimp nauplii. The results indicated that the lichens from Annapurna conservation area (ACA) warrant further research of isolation and characterization of active compounds.

## Abbreviations

ACA: Annapurna conservation area
ASL: Above sea level
BHA: Butylated hydroxyanisole
DCM: Dichloromethane
DPPH: 2,2-Diphenyl-1-picrylhydrazyl;
I: Inactive
IC_50_: 50% inhibition concentration
M: Moderate
mM: Millimolar
NA: Nutrient agar
NB: Nutrient broth
PDA: Potato dextrose agar
S: Strong
TLC: Thin layer chromatography
UV: Ultraviolet
W: Weak
